# The anatomic basis of leptomeningeal metastasis

**DOI:** 10.1084/jem.20212121

**Published:** 2024-03-07

**Authors:** Morgan E. Freret, Adrienne Boire

**Affiliations:** 1 Department of Radiation Oncology, Memorial Sloan Kettering Cancer Center, New York, NY, USA; 2 Department of Neurology, Human Oncology and Pathogenesis Program, Brain Tumor Center, Memorial Sloan Kettering Cancer Center, New York, NY, USA

## Abstract

Leptomeningeal metastasis (LM), or spread of cancer to the cerebrospinal fluid (CSF)–filled space surrounding the central nervous system, is a fatal complication of cancer. Entry into this space poses an anatomical challenge for cancer cells; movement of cells between the blood and CSF is tightly regulated by the blood–CSF barriers. Anatomical understanding of the leptomeninges provides a roadmap of corridors for cancer entry. This Review describes the anatomy of the leptomeninges and routes of cancer spread to the CSF. Granular understanding of LM by route of entry may inform strategies for novel diagnostic and preventive strategies as well as therapies.

## Teaser

Leptomeningeal metastasis (LM), or spread of cancer to the cerebrospinal fluid (CSF) and linings of the brain, is a fatal complication of cancer. In this Review, Freret and Boire describe the anatomy of the leptomeninges and routes of cancer spread to the CSF, highlighting therapeutic opportunities.

## Introduction

Spread of cancer cells to the cerebrospinal fluid (CSF)–filled leptomeninges surrounding the central nervous system (CNS), or leptomeningeal metastasis (LM), represents a neurologically devastating expression of cancer and is among the most underserved therapeutic targets in the CNS (reviewed in Le Rhun et al., 2013). Approximately 5–10% of patients with solid tumors, most commonly breast cancer, lung cancer, and melanoma, develop LM (Brower et al., 2016; Clarke et al., 2010; Wasserstrom et al., 1982). LM also occurs in hematological malignancies and primary CNS tumors (Chang et al., 1969; Meyer et al., 1980; Stewart et al., 1981; Herman et al., 1979).

Patients with LM present with myriad neurological symptoms, reflecting the large neuroanatomic space encompassed by the leptomeninges. These symptoms may include headache, visual change, weakness, constipation, urinary retention, altered mental status, loss of consciousness, and seizure (Pan et al., 2018). On neurologic examination, these symptoms may manifest with papilledema, cranial neuropathies, upper or lower motor neuron pattern weakness, and cauda equina or conus medullaris syndromes. As is typical for a multifocal process, diagnosis can be challenging and is based on clinical features, radiographic studies, and CSF sampling (Le Rhun et al., 2023). LM prognosis is extremely poor with survival limited to 6–8 wk without LM-directed therapy (reviewed in Scott and Kesari, 2013). Despite treatment, prognosis remains poor, with survival ranging from 2 to 10 mo with therapy in modern series (Harstad et al., 2008; de Azevedo et al., 2011; Morris et al., 2012; Kuiper et al., 2015; Niwińska et al., 2015; Brastianos et al., 2020; Yang et al., 2022). Treatment options include systemic therapy; intrathecal therapy; and radiotherapy including whole-brain (Morris et al., 2012), focal spinal (Glantz et al., 1995), and proton craniospinal (Yang et al., 2020, 2022) approaches.

Once within the CSF space, cancer cells may grow in suspension (i.e., free-floating in the CSF) and/or attached to the leptomeninges that coat the brain and spinal cord (Remsik et al., 2020). Commensurately, LM encompasses heterogeneous clinical and radiographic subtypes, with type I LM indicating diagnosis verified cytologically or histologically (e.g., positive CSF cytology) and type II LM indicating diagnosis based on clinical findings and/or neuroimaging only (Le Rhun et al., 2023). On magnetic resonance imaging (MRI), LM may exhibit linear (type A), nodular (type B), mixed (type C), or normal (i.e., absence of MRI abnormality, type D) growth patterns (Le Rhun et al., 2023). Limited retrospective data suggest that the growth pattern in LM (i.e., free-floating versus adherent) may have prognostic significance (Remsik et al., 2020).

Stephen Paget’s “seed and soil” hypothesis proposed that cancer metastasis requires favorable interactions between the metastatic tumor cell (seed) and the organ microenvironment (soil) (Paget, 1889). Considered in this context, the leptomeningeal space is perhaps unique among tumor microenvironments in presenting two significant challenges to cancer metastasis. First, similar to the blood–brain barrier that surrounds the brain parenchyma, communication between the CSF and systemic circulation is tightly regulated by fibroblastoid and epithelial cell barriers (the arachnoid–CSF and blood–CSF barriers, respectively) (reviewed in Abbott et al., 2010). Second, in the absence of cancer, the CSF itself is pauci-cellular and lacks proteins, glucose, and mitogens (Fishman, 1980). Understanding the anatomical routes by which cancer cells infiltrate the leptomeninges, as well as the factors driving this invasion against an apparent nutritional gradient, remain pressing unanswered questions in cancer biology.

Recent studies, propelled by a growing number of syngeneic and xenograft models of LM, have begun to uncover the anatomical routes and molecular and cellular mechanisms mediating cancer cell entry into the leptomeninges. This Review describes the anatomy of the leptomeninges, highlighting sites of anatomical vulnerability, and seeks to catalog our understanding to date of the potential routes by which cancer cells invade the CSF. Cancer infiltration of the CSF is a prerequisite to LM; this step highlights a new understanding of the anatomic and immunologic relationships between the leptomeninges and the larger organism. The subsequent steps in the metastatic cascade that enable cancer cell growth within the leptomeninges are essential questions to the field outside the scope of the present review.

## Anatomy of the leptomeninges

The meninges that surround the brain and spinal cord comprise three layers (reviewed in Weller et al., 2018). The outermost layer is the fibrous dura mater (pachymeninx) located beneath the skull. The dura mater contains endosteal and meningeal layers between which run the meningeal lymphatic vessels as well as fenestrated blood vessels (Mecheri et al., 2018). Unlike capillaries of the blood–brain barrier, these fenestrated blood vessels allow free access of blood components into the dural stroma. As they are freely served by the systemic circulation, the dura therefore reside outside the blood–brain barrier. The leptomeninges reside beneath the dura and consist of the arachnoid and pia mater (Alcolado et al., 1988; Oda and Nakanishi, 1984). The arachnoid is a fibroblastoid cell layer, joined by tight junctions, that is laminated against the dura mater (Nabeshima et al., 1975). Between the arachnoid and pia is the subarachnoid space containing the CSF. The cerebral vessels course through this space en route to the parenchymal tissues of the CNS. Importantly, these vessels, in contrast to dural blood vessels, are characterized by endothelial cell tight junctions conferring barrier properties (Møllgård et al., 2017). The pia comprises a monolayer of fibroblastoid cells (Remsik et al., 2021) connected by desmosomes and gap junctions (Nabeshima et al., 1975). The pia layers atop the glia limitans. The glia limitans basement membrane is formed by interactions between astrocytic endfeet joined by gap junctions (Nabeshima et al., 1975). Between the pia and glia limitans resides the subpia, containing collagen fibers and blood vessels (Zhang et al., 1990); this potential space may enlarge in pathologic conditions.

CSF is produced by the choroid plexuses, specialized structures consisting of an epithelial cell layer, connected by tight junctions, that surrounds fenestrated capillaries carrying blood from the peripheral circulation (Hurley et al., 1981; reviewed in Lun et al., 2015). The choroid plexuses reside within the CSF-filled ventricles. The choroid plexus epithelium represents a polarized secretory epithelium with apical (ventricular) and basolateral (blood-facing) membranes. Among its many functions, the choroid plexus epithelium is responsible for unidirectional transport of ions, including Na^+^, Cl^−^, and HCO3^−^, into the ventricles, which in turn generate an osmotic gradient that provides a driving force for the secretion of water molecules into the ventricles (and thereby production of CSF) in an aquaporin-1–dependent manner (Oshio et al., 2005). In health, the CSF is pauci-cellular, hypoxic, and low in nutrients such as glucose (reviewed in Fishman, 1980).

## CSF barriers

**Figure 1.**
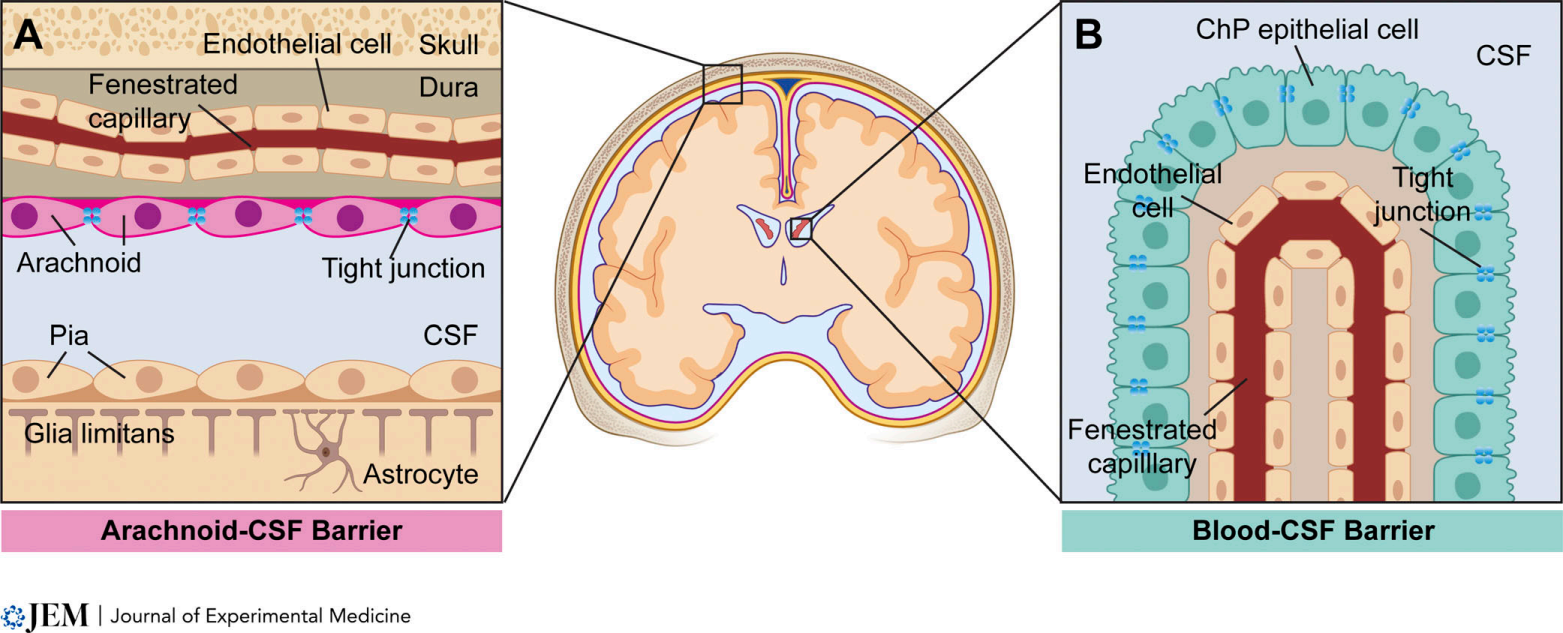
**CSF barrier systems. (A)** The arachnoid–CSF barrier is formed by arachnoid fibroblast-like cells joined by tight junctions, which separate systemic blood within fenestrated dural capillaries from subarachnoid CSF. **(B)** The blood–CSF barrier is formed by choroid plexus (ChP) epithelial cells joined by tight junctions, which separate systemic blood within fenestrated choroidal capillaries from ventricular CSF. Created with https://BioRender.com.

**Figure 2.**
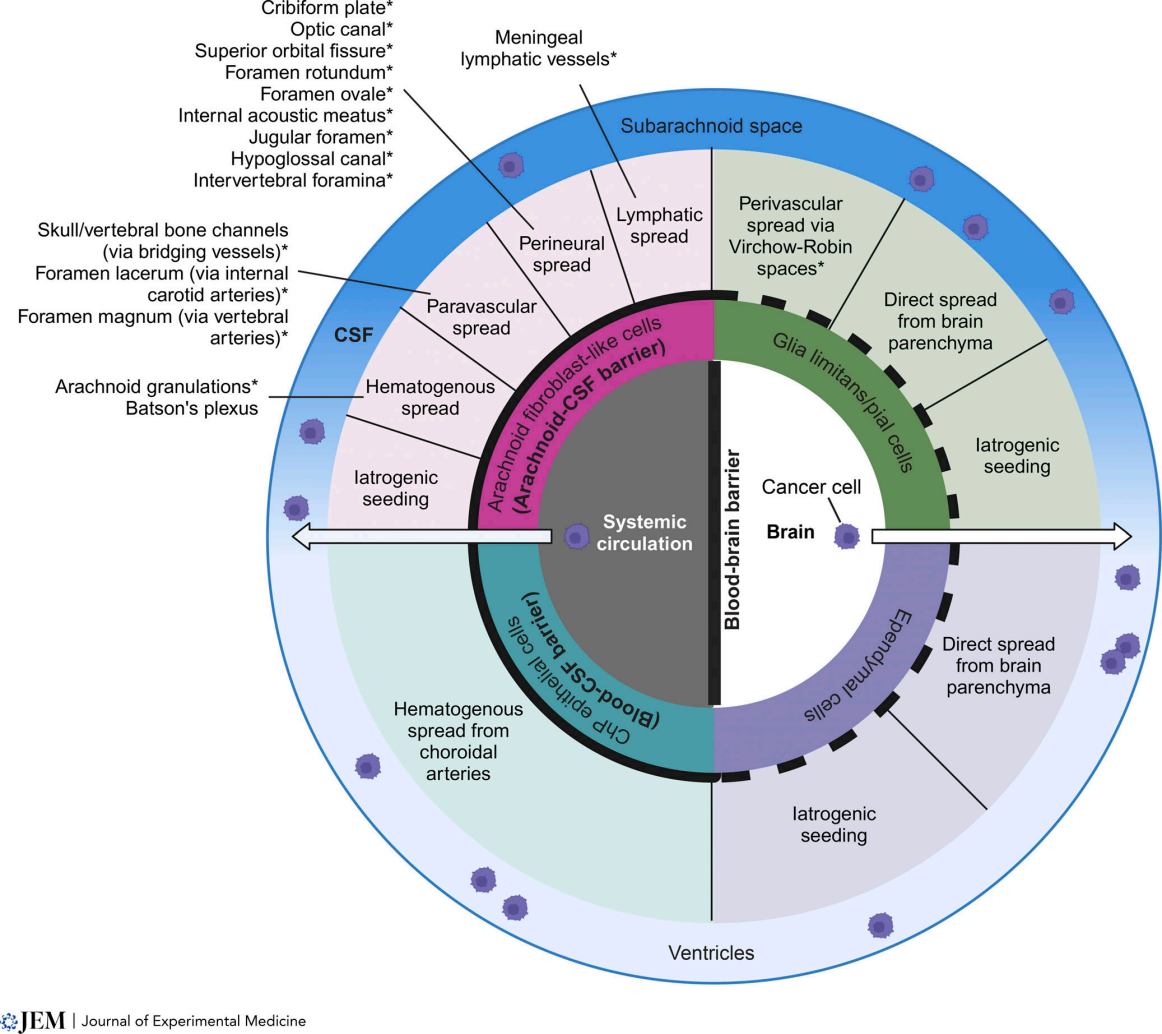
**Overview of the anatomical basis of LM.** This chart represents the possible anatomical routes of cancer spread to the leptomeninges from the systemic circulation or brain. In this concentric circle representation, from inside to outside, circles represent (1) the extracranial site from which cancer originates (e.g., systemic circulation), (2) the cell types comprising each CSF barrier system, (3) anatomical mechanisms of spread, and (4) the CSF, with the upper half representing the subarachnoid space and the bottom half the ventricles. Tight junction–bound barriers are denoted with a thick solid black line (i.e., arachnoid–CSF barrier, blood–CSF barrier, and blood–brain barrier), while interfaces lacking tight junctions are denoted with thick black dashed lines. Asterisk (*) denotes proposed routes of CSF efflux. ChP, choroid plexus. Created with https://BioRender.com.

An anatomical understanding of the leptomeninges highlights the two major barriers separating the CSF from the systemic circulation: (1) the arachnoid–CSF barrier, formed by the tight junctions connecting arachnoid fibroblast-like cells, separating the fenestrated dural blood vessels from the subarachnoid space (Fig. 1 A), and (2) the blood–CSF barrier, formed by choroid plexus epithelial cell tight junctions, separating the fenestrated choroidal arteries from the ventricles (Fig. 1 B) (reviewed in Abbott et al., 2010). Complement C3 generated by cancer cells can impair the function of these choroid plexus epithelial cell tight junctions, opening the blood-CSF–barrier (Boire et al., 2017). A recent study highlighted the possibility that the vascular endothelium within the choroid plexus may close during systemic inflammation (Carloni et al., 2021). However, the functional relevance of this endothelial closure remains to be discovered.

The interface separating the CSF from the brain parenchyma within the ventricular system, the CSF–brain interface, is formed by ependymal cells that line the subventricular zone (reviewed in Saunders et al., 2012). The inner CSF–brain interface contributes to diffusion restriction between the CSF and interstitial fluid (ISF) of the brain parenchyma during development but in the adult has minimal barrier properties (Møllgård et al., 1987; Whish et al., 2015; Liddelow et al., 2012). An exception to this rule are the circumventricular organs (CVOs), sensory structures surrounding the third and fourth ventricles, which lack a true blood–brain barrier and are lined by specialized non-ciliated polarized ependymoglial cells, termed tanycytes. Tanycytes are linked by tight junctions and therefore create a barrier that controls diffusion between the CSF and the brain CVOs (Langlet et al., 2013). Discussed in detail later, the outer CSF–brain interface is characterized by a brain glymphatic system that readily permits exchange of CSF and brain ISF (Iliff et al., 2012; Xie et al., 2013).

In sum, the CSF, which is encased by the brain ventricular system and subarachnoid space, represents a highly privileged anatomical compartment. Entry of immune cells and plasma components is strictly regulated by the choroid plexus epithelial cells at the blood–CSF barrier and by the arachnoid at the arachnoid–CSF barrier. CSF and ISF are freely interchanged between the CSF and brain parenchyma in many regions, whereas the CVOs are characterized by a CSF–brain barrier formed by tanycyte tight junctions. How cancer cells breach these barriers to enter the CSF—and further, why they migrate against a nutritional gradient to do so—is an essential question with relevance to our understanding of LM pathogenesis and efforts to treat and prevent it.

## CSF drainage

The anatomical routes of CSF drainage from the subarachnoid space to the systemic circulation represent potential channels for metastasis to the leptomeninges. CSF produced by the choroid plexuses flows from the lateral ventricles via the foramina of Monro to the third ventricle, and then to the fourth ventricle via the aqueduct of Sylvius. The lateral and medial apertures of the fourth ventricle, termed the foramina of Luschka and Magendie, respectively, carry CSF from the fourth ventricle to the subarachnoid space. Multiple routes of CSF egress from the subarachnoid space to the periphery have been proposed (reviewed in Proulx, 2021), with variable strengths of evidence for each.

Traditionally, it has been suggested that the arachnoid granulations and villi, macroscopic and microscopic outgrowths of the arachnoid, respectively, were the major sites of CSF removal from the subarachnoid space (Boulton et al., 1996; Tripathi, 1974; Welch and Pollay, 1963; Weed, 1914; Key and Retzius, 1876). In this pathway, CSF was thought to flow via the arachnoid villi to the dural venous sinuses, which are channels located between the endosteal and meningeal layers of the dura that drain venous blood from the brain parenchyma and skull into the internal jugular veins (Gray, 2023). More recently, the relative importance of the arachnoid granulations and villi in CSF drainage has been questioned (Biceroglu et al., 2012). By contrast, efflux along perineural, brain perivascular (“glymphatic”), and meningeal lymphatic routes have been proposed to constitute the key removal pathways for CSF.

CSF effluxes from the subarachnoid space to the lymphatic circulation via perineural routes involving both cranial (Lüdemann et al., 2005) and spinal nerves (Grundy, 1962; Edsbagge et al., 2004; Bradbury and Lathem, 1965; Ma et al., 2019). The subarachnoid space extends sleeve-like along cranial and spinal nerves, terminating when the arachnoid fuses with the multilayered connective tissue ensheathing nerve fascicles (perineurium). Ultrastructural studies demonstrate the continuity of the subarachnoid space with the endoneurial extracellular space of spinal nerve roots (Haller et al., 1972). Tracer-based studies show that endoneurial extracellular spaces of both cranial and spinal nerve roots are sites of pressure-driven egress of subarachnoid CSF (Pettersson, 1993). For example, extensions of the subarachnoid space that track along olfactory nerves traverse the cribriform plate and drain CSF to the deep cervical lymph nodes (Bradbury and Cole, 1980; Bradbury and Westrop, 1983; Bradbury et al., 1981; Kida et al., 1993).

The recently discovered glymphatic system, comprising brain perivascular channels (or Virchow-Robin spaces), is a waste clearance system permitting CSF exchange between the brain parenchyma and the subarachnoid space. Anatomically, the cerebral arteries that course along the cortical surface of the brain converge on pial arteries located within the subarachnoid space and subpia (Zhang et al., 1990). Pial arteries enter the brain parenchyma to become penetrating arterioles (Prince and Ahn, 2013). Surrounding these penetrating arterioles are the perivascular Virchow-Robin spaces, with an inner wall facing the arteriole that is bordered by a pial cell membrane and an outer wall bordered by the glia limitans. The CSF-filled Virchow-Robin spaces are continuous with the basement membrane that surrounds the brain parenchymal capillaries. This basement membrane is a thin sheet of extracellular matrix through which CSF can readily flow to reach the perivenous spaces within the brain parenchyma (reviewed in Jessen et al., 2015). Early work employing horseradish peroxidase injections into the subarachnoid space of cats and dogs showed that CSF exits the subarachnoid space via the Virchow-Robin spaces to reach the basement membrane (Rennels et al., 1985, 1990). More recent work using *in vivo* imaging and intracisternal injections of fluorescently labeled tracers confirmed and extended these studies, revealing that subarachnoid CSF that enters the brain parenchyma via the perivascular pathway can intermix with brain ISF and exit the brain via perivenous channels without crossing the endothelial cell layer (blood–brain barrier) of these vessels (Iliff et al., 2012; Xie et al., 2013). Thus, the brain perivascular pathway serves as a conduit by which CSF can exit the subarachnoid space to penetrate the brain parenchyma proper.

The meningeal lymphatic vessels (Cserr et al., 1992) that line the venous sinuses are another important CSF drainage route. Findings show that both dorsal (Louveau et al., 2015; Aspelund et al., 2015) and basal (i.e., skull base) (Ahn et al., 2019) meningeal lymphatic vessels transport CSF and immune cells from the subarachnoid space to the deep cervical lymph nodes. Until recently, an open question for the field was whether the brain glymphatic system and meningeal lymphatics worked in concert to regulate CSF homeostasis. Recent work using a combination of pharmacologic, surgical, and genetic approaches to ablate the meningeal lymphatics found that that meningeal lymphatic function is essential for perivascular influx of CSF into the brain and for the exchange of macromolecules between the CSF and ISF (Da Mesquita et al., 2018b). Thus, the brain glymphatics (perivascular flow) and meningeal lymphatics are functionally linked (reviewed in Da Mesquita et al., 2018a), and both serve as major routes of CSF drainage from the subarachnoid space.

Finally, complementary to the above-described routes of CSF drainage, additional pathways play a likely more minor role in CSF removal. For example, CSF may enter the systemic lymphatic circulation via the paravascular spaces surrounding the internal carotid (Casley-Smith et al., 1976; Wang and Casley-Smith, 1989; Szentistványi et al., 1984) and vertebral (Wang and Casley-Smith, 1989) arteries. In addition, dural/skull vascular channels that directly connect the skull and vertebral bone marrow to the subarachnoid space (Herisson et al., 2018; Cai et al., 2019; Yao et al., 2018) facilitate transit of immune cells and removal of CSF (Mazzitelli et al., 2022).

Taken together, modern understanding of leptomeningeal anatomy provides a blueprint for identifying potential conduits of cancer cell entry into the CSF. Specifically, cancer cells must either directly breach one of the barriers separating the CSF from the systemic circulation (e.g., transcellular or paracellular migration across the choroid plexus epithelium), traverse one of the CSF–brain interfaces, or exploit sites of CSF efflux where barrier function is suboptimal (e.g., migrate in retrograde fashion along cranial or spinal nerves).

## Anatomical routes of cancer spread to the leptomeninges

Numerous potential routes of cancer spread to the CSF have been proposed. The route of spread appears to differ by the location of the seeding tumor (i.e., extracranial versus intracranial location) and by cancer histology, although much work remains to elucidate common pathways. In the next sections, we summarize the evidence for each of these potential anatomical routes of spread, drawing from studies in humans and preclinical LM models.

### Routes of spread for extracranial tumors

For extracranial tumors, potential routes of spread to the CSF include: (1) hematogenous spread from the systemic circulation, (2) retrograde spread along cranial and/or spinal nerves, (3) spread from the bone marrow via dura/skull vascular channels, (4) spread via the meningeal lymphatic vessels, and (5) iatrogenic seeding.

#### Hematogenous spread

Cancer cells within the systemic circulation enter the CSF using both arterial and venous pathways. Autopsy series support both the choroid plexuses (blood–CSF barrier) and the arachnoid (arachnoid–CSF barrier) as cancer cell entry points to the CSF space (Kokkoris, 1983; Olson et al., 1974; Gonzalez-Vitale and Garcia-Bunuel, 1976). In the periphery, the valveless vertebral venous plexus (Batson’s plexus) that surrounds the spinal column (Groen et al., 1997) has also been suggested as a route of spread, particularly in cases of malignancy within the pelvis. This is due in part to the proximity of the internal portion of Batson’s plexus (which abuts the dura surrounding the spinal cord) to the spinal subarachnoid space. However, evidence for CSF invasion via Batson’s plexus is limited to autopsy series and case reports (Bubendorf et al., 2000; Geldof, 1997).

Preclinical models of LM arising from melanoma, breast cancer, and non-small cell lung cancer (NSCLC) all demonstrate that cancer cells may reach the CSF from the systemic circulation. For example, Schackert and Fidler developed the first organotropic model of LM, demonstrating that different subpopulations of melanoma cells preferentially metastasized to the leptomeninges, ventricles, and choroid plexuses versus the brain parenchyma after injection into the internal carotid artery (Schackert and Fidler, 1988; Schackert et al., 1990). This work was the first to suggest that cancer cells that colonized the leptomeninges were biologically distinct from brain-metastatic cancer cells. It also provided the first preclinical evidence for a hematogenous route of cancer spread to the leptomeninges. Extending this work, our group developed leptomeningeal derivatives of breast and NSCLC cell lines, which rapidly colonize the leptomeninges after intracardiac injection (Boire et al., 2017; Chi et al., 2020; Remsik et al., 2020). In these models, cancer cells reproducibly colonize the choroid plexuses a few days before they are detectable within the CSF (Boire et al., 2017). This temporal relationship suggests that cancer cells can reach the CSF after first crossing the choroid plexus epithelium. Given that the choroid plexuses serve as a gateway for immune cell trafficking into the CSF (Shechter et al., 2013), an intriguing although untested hypothesis is that cancer cells hijack immune cell mechanisms to transmigrate across this epithelial cell barrier. A critical limitation of current preclinical models of LM is that the route of cancer cell inoculation greatly influences the metastasis pattern in mouse models (reviewed in Lu and Kang, 2007). Thus, injecting cancer cells into the left ventricle and/or internal carotid arteries may artificially favor metastatic spread via the choroid plexuses, given that the choroidal arteries in the lateral ventricles are subserved by the distalmost branch of the internal carotid artery. Future efforts to develop a model system that recapitulates the entire metastatic cascade from start to finish, rather than one that directly introduces cancer cells into the bloodstream, may identify other pathways of CSF entry.

In sum, diverse cancer types may enter the CSF using arterial and/or venous pathways. Preclinical models of LM indicate that crosstalk between cancer cells and the leptomeningeal microenvironment is complex, is likely to be cell type specific, and may underlie distinct anatomical routes of spread, suggesting therapeutic opportunities.

#### Perineural spread

A wide array of cancers can invade the perineural space surrounding cranial, spinal, and peripheral nerves (reviewed in Chen et al., 2019). Nerves secrete neurotransmitters and growth factors that contribute to tumor progression in pancreatic, prostate, breast, and head and neck cancers, among others (reviewed in Monje et al., 2020). Among patients with vertebral metastases and head and neck cancers, autopsy series demonstrate cancer invasion along spinal and cranial nerves, respectively, suggesting a conduit for leptomeningeal seeding (Kokkoris, 1983). Cancer cells can also invade the lymphatics and/or veins within the neurovascular bundle in close proximity to the subarachnoid space (Gonzalez-Vitale and Garcia-Bunuel, 1976). Although preclinical models of perineural spread giving rise to LM are presently lacking, this remains an important area for future investigation.

#### Spread from the bone marrow via dura/skull vascular channels

An alternate route of cancer spread to the CSF is via the bony vascular channels that directly connect the skull and vertebral bone marrow with the subarachnoid space (Herisson et al., 2018; Cai et al., 2019; Yao et al., 2018). These bony channels harbor bridging vessels that drain the brain parenchyma and subserve migration of multiple immune cell types, including myeloid cells, lymphocytes, and neutrophils, from the skull bone marrow to the meninges, CSF, and brain (Herisson et al., 2018; Cai et al., 2019; Yao et al., 2018; Brioschi et al., 2021; Cugurra et al., 2021). Remarkably, preclinical models of acute lymphoblastic leukemia (ALL) reveal that cancer cells can migrate to the CSF along the abluminal surface of bridging vessels as they course through these bony channels (Yao et al., 2018). This migration is mediated in part through interactions between laminin, an extracellular matrix molecule enriched in bridging vessels, and its receptor α6-integrin, expressed by most cases of ALL (Yao et al., 2018). PI3Kδ regulates ALL cell expression of α6-integrin, and mice with ALL xenografts treated with a PI3Kδ inhibitor or α6-integrin–neutralizing antibodies have reduced numbers of ALL cells within the bridging vessels (but not within the bone marrow) as well as reduced blast counts within the CSF. Notably, in contrast to organotropic models of solid tumor LM (Schackert et al., 1990; Boire et al., 2017), the authors found only rare ALL cells within the choroid plexuses. This incisive work is the first to suggest that microenvironment signals may guide cancer cell migratory routes in a cell type–specific manner.

#### Spread along meningeal lymphatic vessels

Outside the CNS, cancer cells exploit lymphatic vessels for metastatic spread (reviewed in Shayan et al., 2006). The network of meningeal lymphatics within the meninges of the brain and spinal cord provides an anatomical link between the CSF and the periphery (Louveau et al., 2015, 2018; Aspelund et al., 2015; Antila et al., 2017). The proximity of the meningeal lymphatic vessels to the fenestrated (permeable) dural blood vessels, which carry peripheral blood, suggests them as a potential conduit for cancer cell entry from the systemic circulation into the CSF. This hypothesis presupposes retrograde cancer spread along a vessel that normally carries fluid and cells away from the subarachnoid space. At this time, a role for these extra-axial meningeal lymphatic vessels in LM initiation and in immune cell trafficking and response to LM remains speculative.

#### Iatrogenic seeding

Although less well described than for intracranial tumors, iatrogenic seeding of the CSF by circulating tumor cells (CTCs) from the peripheral circulation represents another potential route of leptomeningeal infiltration. In the case of hematologic malignancies such as ALL, traumatic lumbar puncture with seeding of the CSF by blast cells is associated with poorer outcomes (Gajjar et al., 2000). In comparison to hematologic malignancies, solid tumors have a substantially lower CTC burden; therefore, iatrogenic seeding of the CSF after traumatic lumbar puncture is likely a much lower frequency event.

#### Insights from the blood–brain barrier in metastasis

Although how cancer affects the structure and function of the blood–CSF barriers remains mostly uncharted territory, insights from studies of the analogous blood–brain barrier provide a useful framework for understanding routes of cancer spread to the CSF. The arachnoid–CSF and blood–CSF barriers tightly regulate cellular and molecular transport between the peripheral circulation and CSF by virtue of their fibroblastoid and epithelial cell layers, respectively, which are connected by tight junctions. The analogous blood–brain barrier is formed by tight junctions connecting endothelial cells and regulates brain parenchymal homeostasis (reviewed in Arvanitis et al., 2020). Cancer metastasis compromises the integrity of the blood–brain barrier (Lyle et al., 2016; Percy et al., 2011; Steeg, 2016; Steeg et al., 2011; Bos et al., 2009), and some have suggested that this disruption is essential to cancer transmigration across the blood–brain barrier into the brain parenchyma (Arvanitis et al., 2020). Both paracellular and transcellular cancer migration routes have been proposed (Arvanitis et al., 2020; Malladi et al., 2016). In light of our understanding of the blood–brain barrier in facilitating cancer invasion of the brain, it is reasonable to suggest that, in a similar fashion, the major routes of cancer invasion of the CSF from the periphery may be via transmigration across its tight junction–bound barrier systems. Supporting this hypothesis is the observation that proposed migration along routes of CSF efflux, as cataloged above, generally requires cancer cells to move against the bulk flow of CSF; one might instead expect cancer to be removed from the CSF space by these routes.

### Routes of spread for intracranial tumors

For intracranial tumors, which comprise both primary CNS tumors (e.g., medulloblastoma) and brain metastases from solid tumors, proposed routes of spread to the CSF include: (1) direct and/or perivascular spread from the brain parenchyma, (2) hematogenous spread from intracranial tumor cells that have accessed the systemic circulation, and (3) iatrogenic seeding.

#### Direct and/or perivascular spread

LM coexists with solid tumor brain metastases in some 50–80% of patients (Clarke et al., 2010; Morris et al., 2012; Umemura et al., 2012; Lara-Medina et al., 2012). This has led to the suggestion that LM may arise due to direct spread from brain parenchymal lesions. Autopsy series provide some support for a model in which cancer cells directly breach the glia limitans/pia of the brain surface and/or the ependyma lining the brain ventricles (Boyle et al., 1980). Furthermore, brain metastasis location (i.e., bordering the CSF space) is associated with LM risk in retrospective studies (Lowe et al., 2022), although this was not born out in a recent metanalysis of 2,105 patients (Tewarie et al., 2021). Dankner, Siegel, and colleagues showed in a recent histopathological series of resected brain metastases that an invasive growth pattern was more likely to coexist with LM at the time of resection (Dankner et al., 2021a). These findings provide correlative support for LM resulting from brain parenchymal lesions.

Preclinical models of brain metastasis likewise suggest that cancer cells in the brain parenchyma can spread to the CSF. For example, LM has been observed after intracardiac injection of brain-metastatic derivatives of both patient-derived breast cancer cells and MDA-MB-231 cells (a human breast cancer cell line); this may be due to secondary invasion of these brain metastases into the leptomeningeal space (Bos et al., 2009). Another study showed that mice injected intracranially with brain-metastatic MDA-MB-231 cells rapidly developed LM in addition to tumors at the injection site (Allen et al., 2014). The authors measured CTCs in the CSF and peripheral blood in a single mouse and found CTCs in both, indicating that tumor cells injected into the brain parenchyma can migrate to both compartments. Commensurately, intracranial injection of patient brain metastasis–derived cancer cells leads to LM in a subset of mice (Dankner et al., 2021a). In both patients and xenografted mice harboring brain metastases, the risk for LM was associated with highly invasive brain parenchymal growth patterns and expression of cold-inducible RNA binding protein (Dankner et al., 2021a). Taken together, these studies strongly suggest that subsets of cancer cells within the brain parenchyma are competent to colonize the CSF.

However, because these models employ intracardiac injection or direct injection into the brain parenchyma (thereby creating a direct track from the brain parenchyma to the leptomeninges), they cannot distinguish whether CSF spread occurs via direct invasion across the glia limitans and/or ependyma or, as suggested by others (Dankner et al., 2021b), via the glymphatic system. In the latter, cancer cells within the perivascular spaces of brain blood vessels (Kienast et al., 2010) invade the subarachnoid space by passing through the basement membrane to reach the Virchow-Robin spaces. As described earlier, the pial border of the Virchow-Robin spaces lacks tight junctions; these spaces therefore directly communicate with the subarachnoid space. Although speculative, a brain-specific perivascular route of cancer spread is compelling anatomically as it involves cancer cells coopting a natural weakness in the CSF’s barrier systems. Notably, cancer spread via brain perivascular spaces, if proven, would suggest the prospect of bidirectional spread, in which cancer cells within the brain parenchyma can seed the CSF and those within the CSF can seed the brain parenchyma.

#### Hematogenous spread

Remarkably, spread from the brain parenchyma to the leptomeninges via the systemic circulation is confirmed by recent work using a combination of parabiosis and medulloblastoma xenografts (Garzia et al., 2018). In this elegant study, a donor mouse underwent cerebellar grafting of human medulloblastoma cells followed by parabiosis surgery to a recipient (sister) mouse. All donor mice developed cerebellar medulloblastoma and LM, and when symptomatic, were sacrificed after the reversal of the joining surgery. In half of the parabiosis pairs, the surviving recipient sister mouse went on to develop LM. Furthermore, overexpression of the chemokine CCL2 was sufficient to drive LM in infrequently metastatic medulloblastoma lines ONS76 and MB002, while short hairpin RNA–mediated knockdown of CCL2 in the highly metastatic D425S medulloblastoma line decreased metastasis (Garzia et al., 2018). In sum, medulloblastoma-CTCs in the peripheral circulation can home to the leptomeninges to form metastases, and the CCL2–CCR2 chemokine axis mediates leptomeningeal dissemination in medulloblastoma. While this study showed that medulloblastoma cells can invade the CSF from the systemic circulation, it does not rule out direct and/or perivascular shedding of medulloblastoma cells from the primary tumor into the CSF.

Given evidence for systemic CTCs from patients harboring other primary brain tumor types, including both adult and pediatric gliomas (Macarthur et al., 2014; Müller et al., 2014; Sullivan et al., 2014; Zaky et al., 2023), the potential for a hematogenous route of CSF seeding—or reseeding, as described below—is a provocative potential pathway for spread of intracranial malignancies.

#### Iatrogenic seeding

Reported rates of LM after brain metastectomy range from 6 to 33% (Jung et al., 2012; van der Ree et al., 1999; Ahn et al., 2012; Suki et al., 2008; Huang et al., 2014). Iatrogenic LM is thought to arise due to microscopic (or in some cases macroscopic) surgical spillage at the time of resection of both brain metastases (van der Ree et al., 1999; Ahn et al., 2012; Jung et al., 2012; Ma et al., 2018) and primary malignant brain tumors (Grabb et al., 1992; Toledano Delgado et al., 2006; Roelz et al., 2015). For example, a case control study of 49 patients treated with stereotactic radiosurgery for brain metastases from NSCLC, breast cancer, or melanoma found that prior surgical resection was associated with 6.5 higher odds of LM (Ma et al., 2018). Findings suggest that the risk for iatrogenic seeding is greatest after piecemeal resection (Ahn et al., 2012; Suki et al., 2008, 2009) and for tumors located within the posterior fossa (Mirimanoff and Choi, 1987; Norris et al., 1997; van der Ree et al., 1999). Ventricular entry during surgical resection also appears to be a risk factor for LM (Roelz et al., 2015; Lowe et al., 2022). Studies investigating iatrogenic LM are limited by their retrospective nature and should be interpreted with caution; numerous large studies have also found no increased risk for LM after surgical resection of intracranial tumors (Cagney et al., 2019), and prospective data are lacking.

## Future directions

Since the first description of leptomeningeal metastasis (Eberth, 1870), there has been growing appreciation for the complex challenges presented by the leptomeningeal microenvironment to cancer cell invasion, growth, and survival. The evidence for stereotyped anatomical routes of LM seeding, summarized in this Review, supports the notion that cancer cells invade the CSF either by exploiting sites of suboptimal CSF barrier function (e.g., routes of CSF efflux) and/or directly crossing barriers using transmigratory pathways employed by other cell types (e.g., immune cells and the choroid plexuses) (Fig. 2). A pressing challenge for the field is to integrate across observations from human histopathological studies and an array of preclinical models to reveal unifying principles that drive invasion and growth within the leptomeninges.

### Tumor type–specific anatomical routes of spread

The metastatic process is non-random, and different tumor types manifest distinct patterns of metastatic spread (organotropism) (Hess et al., 2006). Breast cancer, lung cancer, and melanoma account for nearly 80% of solid tumor LM in some series (Clarke et al., 2010). Among breast cancers, lobular subtype and triple-negative cancers have an increased risk of LM (Abouharb et al., 2014). Among NSCLC, epidermal growth factor mutation (Liao et al., 2015; Li et al., 2018) and possibly also anaplastic lymphoma kinase rearrangement (Gainor et al., 2013) are risk factors for LM. In addition to cancer type and subtype, metastatic organotropism as an organizing principle can be applied to the intrinsic cellular heterogeneity that exists within a tumor. Fidler and colleagues’ experimental metastasis models showed that only a subset of cells derived from a parental cell line are able to metastasize to the leptomeninges (Schackert and Fidler, 1988; Schackert et al., 1990). Paget’s seed and soil hypothesis provides a powerful explanation for this cancer type– and cell–specific tropism for the leptomeninges. As cancer cells disperse throughout the body, metastasis occurs only when cancer cells (seeds) land in a compatible microenvironment (soil).

This explanation for organ-specific metastasis, however, requires further development in the context of a tumor microenvironment safeguarded by tight junction–bearing barrier systems such as the leptomeninges. To what extent does the anatomical proximity of a primary or metastatic lesion to sites of suboptimal CSF barrier function govern likelihood of LM? Or, is migration into the CSF primarily driven by interactions between a cancer cell and the unique cell types and molecules of the anatomical route itself? In the first alternative, cancer seeds are released adjacent to a door opening onto a fenced-in field of CSF soil. The second alternative is akin to the sticky burrs that cover burdock seeds that allow them to cling to the fur of a passing animal for dispersal. Although much work remains, findings in models of leukemia LM suggest that a combination of these two alternatives, complementary and in addition to Paget’s original seed and soil hypothesis, is a meaningful way to understand tumor type–specific leptomeningeal colonization. ALL cells residing in vertebral bone marrow access the CSF via vascular channels that connect the bone marrow and subarachnoid space (alternative 1). This migration is mediated in part by interactions between laminin in the extracellular matrix of these bridging vessels and its receptor α6-integrin, expressed by ALL cells (alternative 2) (Yao et al., 2018). Thus, the anatomical route of entry into the CSF is, to an extent, specified and constrained both by anatomical proximity and by instrinsic features of the cancer cells themselves.

Applying this framework to metastatic solid tumors represents a more daunting challenge. In contrast to ALL, which infrequently occurs at extramedullary sites, solid tumors exhibit myriad growth and metastasis patterns. Despite this complexity, emerging data are beginning to uncover clues into solid tumor-specific CSF invasion. Analyses of cell-free RNA in the CSF of patients with NSCLC LM revealed the presence of transcripts of the NSCLC-associated gene carcinoembryonic antigen family-related cell adhesion molecule 6 (*CEACAM6*) (Li et al., 2021). Importantly, these transcripts represented bonified expression by NSCLC LM cells, and expression of CEACAM6 is both necessary and sufficient to drive cell migration in an array of NSCLC cell lines (Li et al., 2021). This provocative work suggests a potential role for overexpression of CEACAM6 on the surface of NSCLC cells in permitting CSF invasion in a tumor type–specific manner. Specifically, associations between CEACAM6 on the surface of NSCLC cells and cell-surface integrins might perturb cell–cell and/or cell–extracellular matrix interactions associated with specific migratory paths (Duxbury et al., 2004). Differences among solid tumors in leptomeningeal tropism underscore the need for careful study of each type of cancer to uncover the ways in which cancer type and location cooperate to define routes of leptomeningeal access.

Further complicating this challenge is understanding how pretreatment with a wide array of cancer therapies alters cancer’s ability to colonize the CSF. A recent study longitudinally profiled the liquid LM tumor microenvironment before and after immune checkpoint inhibitor therapy to reveal novel T cell responses and clinical correlates (Prakadan et al., 2021). How treatment-related changes in cell populations and gene expression in the premetastatic state perturb environmental signals to drive and/or inhibit CSF spread remains to be determined.

A fruitful approach to decoding tumor type–specific routes of CSF invasion, which has been applied to brain metastasis (and the analogous blood–brain barrier) (Bos et al., 2009; Valiente et al., 2020), is to develop diverse models of LM by cancer type, subtype, and site of injection (e.g., tail vein versus arterial) and to integrate findings across models to arrive at a holistic understanding of how cellular and subcellular properties of heterogeneous cancer types cooperate to give rise to stereotyped patterns of CSF invasion. In a major step toward developing model systems that more faithfully recapitulate human disease, pioneering recent work enabled the first successful in vitro and xenograft models of patient-derived melanoma LM (Law et al., 2022) and breast cancer LM (Fitzpatrick et al., 2023). We are optimistic that these resources can be deployed to better define cancer type–specific routes of anatomical spread. Understanding precisely where different solid cancers enter the CSF space is an ongoing challenge that will require the sustained and collaborative efforts of multiple research groups.

### Molecular targets for LM preventive strategies

Some have suggested that the multiplicity of potential anatomical pathways for cancer cells to enter the CSF renders futile efforts to uncover the cellular and molecular processes underpinning CSF invasion (Dankner et al., 2021b). Rather, research efforts should focus on elucidating the factors that mediate cancer growth once within the subarachnoid space. An alternative interpretation is that the significant anatomical challenge of entering the protected CSF space exerts a strong convergent evolutionary pressure on cancer cells. In such a scenario, metastatic cells from diverse cancer types, including breast cancer, lung cancer, and melanoma, all converge on relatively few shared anatomical route(s) subserved by common microenvironment-derived factors, to subvert the CSF barrier systems to invade the subarachnoid space. We are encouraged by the present evidence of the possibility of identifying broadly shared pathways and mediators of CSF spread across cancer types, and that these pathways and mediators can be blocked therapeutically.

An approach that seeks to prevent LM, rather than to treat it after it has already blossomed, is attractive because of the diffuse nature of cancer spread once within the CSF (cancer cells can rapidly disseminate to coat the surfaces of the brain and spinal cord), which causes significant (generally irreversible) neurological morbidity in patients and presents a substantial therapeutic challenge. The discovery of key cellular and molecular mediators driving cancer migration into the CSF holds the potential to identify high-risk patient groups who may benefit from CNS prophylaxis and/or increased CSF surveillance as well as targets for rational design of therapies to prevent LM. High-risk patient groups might be identified by a combination of cancer- and/or patient-specific factors, for example, molecular profiling of tumors to reveal “high-risk” transcriptional states for leptomeningeal spread or patient-specific polymorphisms in extracellular matrix glycoproteins critical for migration. Depending on a cancer type’s preferred route of CSF invasion, such targeted therapies for LM prevention might, theoretically, be designed to abrogate cancer cell transcytosis, paracellular permeability across a CSF barrier (for example, via integrin-binding tumor-homing peptides), or cancer cell adhesion to a critical endothelial and/or epithelial cell layer.

### The leptomeninges as a cancer sanctuary site

A related key question is raised by major discoveries in the last decade related to CNS border-associated immune cell composition and trafficking. These discoveries include the functional significance of the brain perivascular spaces (Iliff et al., 2012; Xie et al., 2013), the meningeal lymphatic vessels (Louveau et al., 2015; Aspelund et al., 2015), and the ossified vascular channels directly connecting the skull and vertebral bone marrow to the subarachnoid space (Herisson et al., 2018; Cai et al., 2019; Yao et al., 2018). The brain glymphatic system provides a clear pathway from the brain parenchyma to the subarachnoid space. Considered in sequence with various routes connecting the subarachnoid space and the systemic circulation (e.g., meningeal lymphatic vessels), this work raises the possibility of cancer cell cycling among the CSF, brain, and systemic compartments (intercompartmental cycling). A key feature of LM is the ability of cancer cells to grow in suspension in the CSF (CTCs). The extent to which, after LM is established, CTCs in the CSF use these intra- and extracranial pathways to cycle among the CSF, brain, and periphery is unknown; however, it suggests a potential anatomical explanation for resistance to systemic and intrathecal therapies. Whether the CSF serves as a meaningful sanctuary site for cancer cells in solid tumor malignancies, whether CSF CTCs contribute to “self-seeding” of parenchymal lesions within the brain and periphery, and the extent to which intercompartmental cycling occurs across various cancer types are important, open questions for the field.

## Concluding remarks

LM is a devastating complication of metastatic cancer that warrants vigorous clinical, translational, and biological investigation. By identifying the key anatomical routes of LM seeding, as well as the molecular factors mediating these cellular interactions, we will uncover druggable targets expressed by cancer cells and their microenvironmental signaling partners. Such a strategy underscores the power of cancer as a living probe that can be used to discover key biomolecules, cellular properties, and anatomical routes with relevance to both normal biological systems and malignancy. Armed with this knowledge, we will be better positioned to develop rational individualized therapies to prevent and treat LM and improve patient outcomes.
